# CAR T- cell therapy provides an opportunity for further consolidation treatment for relapsed or refractory adult Burkitt lymphoma patients

**DOI:** 10.3389/fonc.2025.1566938

**Published:** 2025-05-23

**Authors:** Rui Liu, Fan Yang, Lixia Ma, Yuelu Guo, Miaomiao Cao, Zhonghua Fu, Biping Deng, Qinlong Zheng, Chen Chen, Danyang Li, Xiaoyan Ke, Kai Hu

**Affiliations:** ^1^ Department of Lymphoma and Myeloma Research Center, Beijing GoBroad Hospital, Beijing, China; ^2^ Cytology Laboratory, Beijing GoBroad Boren Hospital, Beijing, China; ^3^ Department of Medical Laboratory, Beijing GoBroad Boren Hospital, Beijing, China

**Keywords:** Burkitt lymphoma (BL), car-t, Allo-HSCT, allogeneic hematopoietic stem cell transplantation, auto-HSCT, mutation - genetics, IL-6

## Abstract

**Background:**

Relapsed or refractory (R/R) Burkitt lymphoma (BL) in adults is aggressive and lacks standardized salvage options. Data on the efficacy and safety of chimeric antigen receptor T (CAR-T) cell therapy in this population remains limited.

**Methods:**

We retrospectively analyzed 25 adult patients with relapsed or refractory Burkitt lymphoma who received CAR T-cell therapy. Clinical data, treatment responses, and survival outcomes were collected from medical records. Bridging therapy and lymphodepleting regimens varied based on disease status. Treatment-related toxicities and CAR-T expansion were monitored. Primary endpoints included efficacy, safety, and survival. Risk factors associated with treatment outcomes were explored using univariate analyses.

**Results:**

One month objective response rate (ORR) was 52%(13/25)(95%CI: 31.3–72.2), with a complete response rate (CRR) of 28% (7/25). Sixteen patients (64%) received sequential consolidation therapy including 9 who received a second CAR-T infusion, and 7 who proceeded to autologous or allogeneic hematopoietic stem cell transplantation. The median follow-up time was 26.10 months (range 14.50-57.17). The median OS was 5.49 months(95%CI 1.74-9.25), and the median PFS was 2.96(95%CI 1.62-4.3)months. At last follow-up(2024-08-22), 28% achieved disease-free survival, with one patient disease-free for 5 years.

**Conclusions:**

CAR-T therapy shows promising activity in relapsed/refractory Burkitt lymphoma, but its effectiveness is limited by short response duration. High-risk features may predict poor outcomes, and a higher number of long-term survivors were observed in patients who received transplant sequential consolidation. However, due to the small sample size, larger studies are needed to validate these findings.

## Introduction

Burkitt lymphoma (BL) is a highly aggressive subtype of B-cell non-Hodgkin lymphoma characterized by rapid proliferation and a high propensity for extranodal involvement, including the bone marrow and central nervous system (CNS) (1). It is strongly associated with MYC gene rearrangements, which drive uncontrolled cellular proliferation (2, 3). First-line treatment typically involves dose-intensive, short-cycle chemotherapy regimens, such as CODOX-M/IVAC, Hyper-CVAD, or DA-EPOCH-R, specifically tailored to target BL’s rapid proliferation and achieve durable remission. These regimens have demonstrated excellent outcomes in newly diagnosed BL, with five-year overall survival (OS) rates of 70–91% (4–12). In contrast, standard regimens like R-CHOP, which are effective in diffuse large B-cell lymphoma (DLBCL), are not recommended for BL due to their lower efficacy in this highly aggressive disease (6).Despite these encouraging results in newly diagnosed patients, the prognosis for relapsed or refractory (R/R) BL remains extremely poor. Patients with refractory disease or early relapse (within six months) often show resistance to conventional salvage chemotherapy, leaving limited effective options (13). Salvage regimens such as R-ICE, GDP, or even EPOCH, while occasionally providing transient disease control, rarely achieve long-term remission. For eligible patients, high-dose chemotherapy followed by autologous stem cell transplantation (ASCT) may offer some benefit, but outcomes are generally suboptimal (14, 15). Furthermore, the role of targeted agents, such as BTK inhibitors and PI3K inhibitors, remains largely investigational and has not yet been established as standard therapy in R/R BL. In recent years, chimeric antigen receptor T-cell (CAR-T) therapy has revolutionized the treatment landscape for relapsed/refractory large B-cell lymphoma (LBCL), demonstrating durable responses and five-year survival rates of up to 40% in some studies (16, 17). However, evidence supporting the efficacy of CAR-T therapy in BL remains sparse. BL’s unique biological characteristics, including its rapid doubling time, high tumor burden, and distinct microenvironment, may pose additional challenges to CAR-T efficacy. Most available data on CAR-T in BL come from isolated case reports or small series, highlighting a critical unmet need for systematic evaluation (18–20). In this study, we conducted a retrospective analysis of patients with R/R BL treated with CAR-T therapy, including the sequential or combined use of additional targeted CAR-T products and/or hematopoietic stem cell transplantation (HSCT). Our primary objectives were to assess the efficacy, safety, and long-term survival outcomes of CAR-T therapy in this patient population. Additionally, we aimed to identify factors influencing treatment responses and explore opportunities to optimize therapeutic strategies for this challenging disease.

## Method

### Patients and study design

We conducted a retrospective analysis of 25 patients with relapsed or refractory (R/R) Burkitt lymphoma who received CAR-T cell therapy at Beijing GoBroad Hospital between December 13, 2018, and May 31, 2023.The inclusion criteria were as follows: (1) age between 18 and 80 years; (2) diagnosed with R/R Burkitt lymphoma, where refractory disease was defined as failure to achieve at least partial remission after first-line therapy or disease progression during treatment, and relapse was defined as the reappearance or progression of the disease after an initial response or remission; (3) MYC rearrangement or translocation detected by Fluorescence *in situ* hybridization (FISH); The trial protocol was approved by the Ethics Committee of Beijing GoBroad Hospital in accordance with the Declaration of Helsinki. The study participants were derived from two clinical trials (ChiCTR2200058972 and ChiCTR2100055062) with ethics approval number WZ2024-001-001. All participants or their legal guardians provided written informed consent.

### CAR-T cell manufacturing

All patients underwent lymphocyte apheresis and CAR-T cell preparation, which were conducted in the laboratory of Beijing GoBroad Hospital. Peripheral blood mononuclear cells (PBMNCs) were isolated from eligible patients, and CD3+ T lymphocytes were separated using antigen-coated immunomagnetic beads. The detailed preparation process has been described in previous studies (21–24).

### Bridging and lymphodepletion therapy

Prior to CAR-T cell infusion, bridging therapy was permitted. Bridging therapy was defined as lymphoma-directed treatment administered between leukapheresis and lymphodepletion (LD). The choice of bridging therapy depended on tumor burden and included drugs not previously used or those without resistance to guide an individualized regimen combining chemotherapy and targeted molecular agents.

### CAR-T cell infusion and monitoring

Patients were hospitalized for at least 14 days after CAR-T infusion to monitor cytokine release syndrome (CRS), immune effector cell-associated neurotoxicity syndrome (ICANS), hematologic toxicity, and infections. Hematologic toxicity was evaluated based on levels of neutrophils, platelets, and hemoglobin within the first month post-infusion and graded according to CTCAE 5.0. CRS and ICANS were assessed and graded following ASTCT consensus criteria. Corticosteroids and/or tocilizumab were administered based on the severity of CRS/ICANS and patient tolerance.

### CAR-T expansion and response evaluation

CAR-T cell expansion was monitored using flow cytometry and PCR on days 3, 7, 11, 14, 21, 30, 60, and 90 after infusion. Treatment efficacy was assessed one month after CAR-T infusion based on imaging evaluations following the Lugano criteria, and flow cytometry was employed for bone marrow and cerebrospinal fluid evaluation.

### Consolidation and follow-up

Consolidative treatments, if needed, included additional CAR-T therapies targeting other antigens, autologous or allogeneic hematopoietic stem cell transplantation, targeted drugs, multi-agent chemotherapy, or radiotherapy. The remission rates and long-term survival outcomes of patients receiving CAR-T therapy and subsequent integrated treatments were analyzed. Progression-free survival (PFS) was defined as the time from the first CAR-T infusion to disease progression, death, or last follow-up, while overall survival (OS) was defined as the time from the first CAR-T infusion to death or last follow-up.

### Data collection

Baseline characteristics, treatment-related toxicities, clinical responses, and survival outcomes were extracted from the REDCap (Research Electronic Data Capture) database based on detailed data extracted from patients’ medical records.

### Statistical analysis

All statistical analyses were performed using SPSS 26.0,SAS 9.4 and GraphPad Prism 9.0. Continuous variables were reported as medians and ranges, while categorical variables were presented as frequencies and percentages. Median progression-free survival (PFS) and overall survival (OS), as well as 1-year PFS and OS rates and their 95%CI were estimated using the Kaplan-Meier method. Cox regression were conducted to identify prognostic factors for PFS and OS.

## Results

### Baseline demographic characteristics

Of the 25 patients, 72% (18/25) were male, with a median age of 36 years (range 19–65). One patient had HIV, two had EBV, five had bone marrow involvement, and two had central nervous system involvement. Advanced-stage disease (stage III–IV) was observed in 88% (22/25), and 96% (24/25) had received ≥3 lines of prior therapy. The cohort included 64% (16/25) refractory cases and 36% (9/25) relapsed cases, with 44% (4/9) relapsing within six months. The median time from diagnosis to admission was 0.57 months (range 0.21–7.27), and nine patients had tumor sizes >10 cm. Induction therapies included R ± CHOP (15/25), Hyper-CVAD-AB (4/25), EPOCH (5/25), and ABVD (1/25). Two patients had a history of autologous transplantation, and two had undergone allogeneic transplantation. Nine out of 25 patients had lactate dehydrogenase levels(LDH) exceeding three times the normal value at the time of lymphocyte collection. Next-generation sequencing (17/25) revealed frequent mutations in TP53 (14/17), ID3 (8/17), MYC (8/17), and CCND3 (5/17). Bridging therapy was administered to 19 patients. 12 patients received platinum-based chemotherapy regimens, and 7 patients received anthracycline-based regimens. Notably, 10 patients received a regimen incorporating polatuzumab vedotin (POLA). The median number of bridging therapy cycles was one (range: 1–3).Five achieved partial response (PR), three had stable disease (SD), and 11 showed disease progression (PD). Lymphodepletion regimens varied among patients: 18 received fludarabine and cyclophosphamide (FC) and 7 patients did not receive additional lymphodepletion because their absolute lymphocyte count had already dropped below 0.1 × 10⁹/L following bridging chemotherapy. The baseline clinical characteristics are summarized in (**Table 1**).

**Table 1:** Characteristics of patients at baseline.

Parameters	Patients
Sex (male/female)	7(28%)/18(72%)
Age, median [range]	36 [19–65]
Diagnosis_to_admission(year), median[range]	0.57 [0.21-7.27]
Ann Arbor stage	
Stage I-II	3/25(12%)
Stage III-IV	22/25(88%)
IPI risk	
low risk	2/25(8%)
low-intermediate risk	12/25(48%)
high-intermediate risk	7/25(28%)
high risk	4/25(16%)
ECOG	
0-1	16/25(64%)
2-4	9/25(36%)
Bulky disease>10cm	9/25(36%)
LDH > 750 (U/L)	9/25(36%)
BM involved	5/25(20%)
CSF involved	2/25(8%)
NGS-TP53	14/17(82%)
NGS-ID3	8/17(47%)
NGS-CCND3	5/17(29%)
NGS-MYC	8/17(47%)
Previous chemotherapy cycles, median [range]	7 [3-16]
Previous ASCT therapy	2/25(8%)
Previous allo-HCT therapy	2/25(8%)

### Response

**Figure 1:**
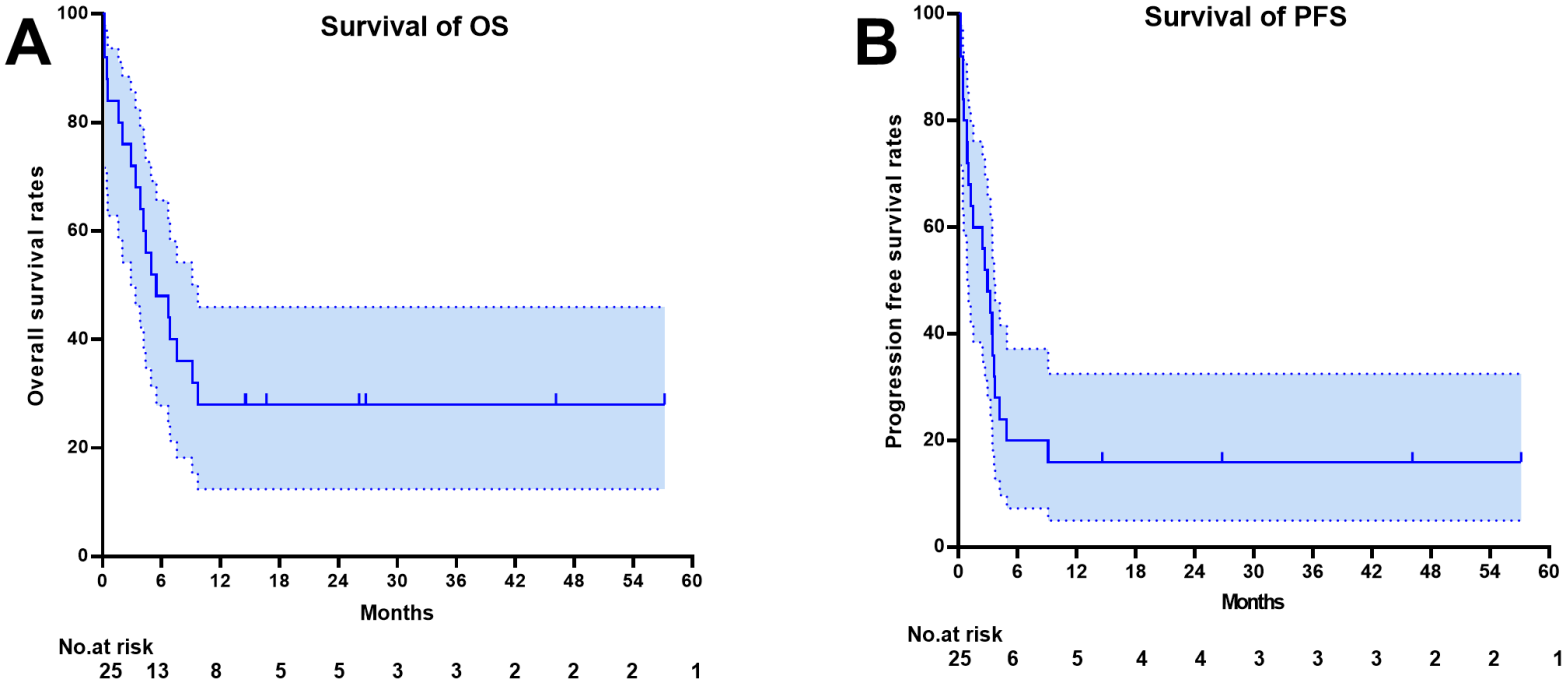
Survival outcome depicted by the Kaplan-Meier curve. **(A)** Overall Survival **(B)** Progression Free Survival.

One month after CAR-T therapy, the objective response rate (ORR) was 52% (95%CI: 31.3–72.2)(13/25), with a complete response rate (CRR) of 28% (7/25). The median follow-up time was 26.10 months (range 14.50-57.17 months). The median overall survival (OS) for the 25 patients was 5.49 months (95% CI 1.734 – 9.25), and the median progression-free survival (PFS) was 2.96 months (95% CI 1.62 – 4.30). The estimated 1-year progression-free survival and overall survival rates were 16%(95%CI 5.02-32.54) and 28%(95%CI 12.42-45.98), respectively. As of the last follow-up, 28% (7/25) were in disease-free survival (**Figure 1**).

**Figure 2:**
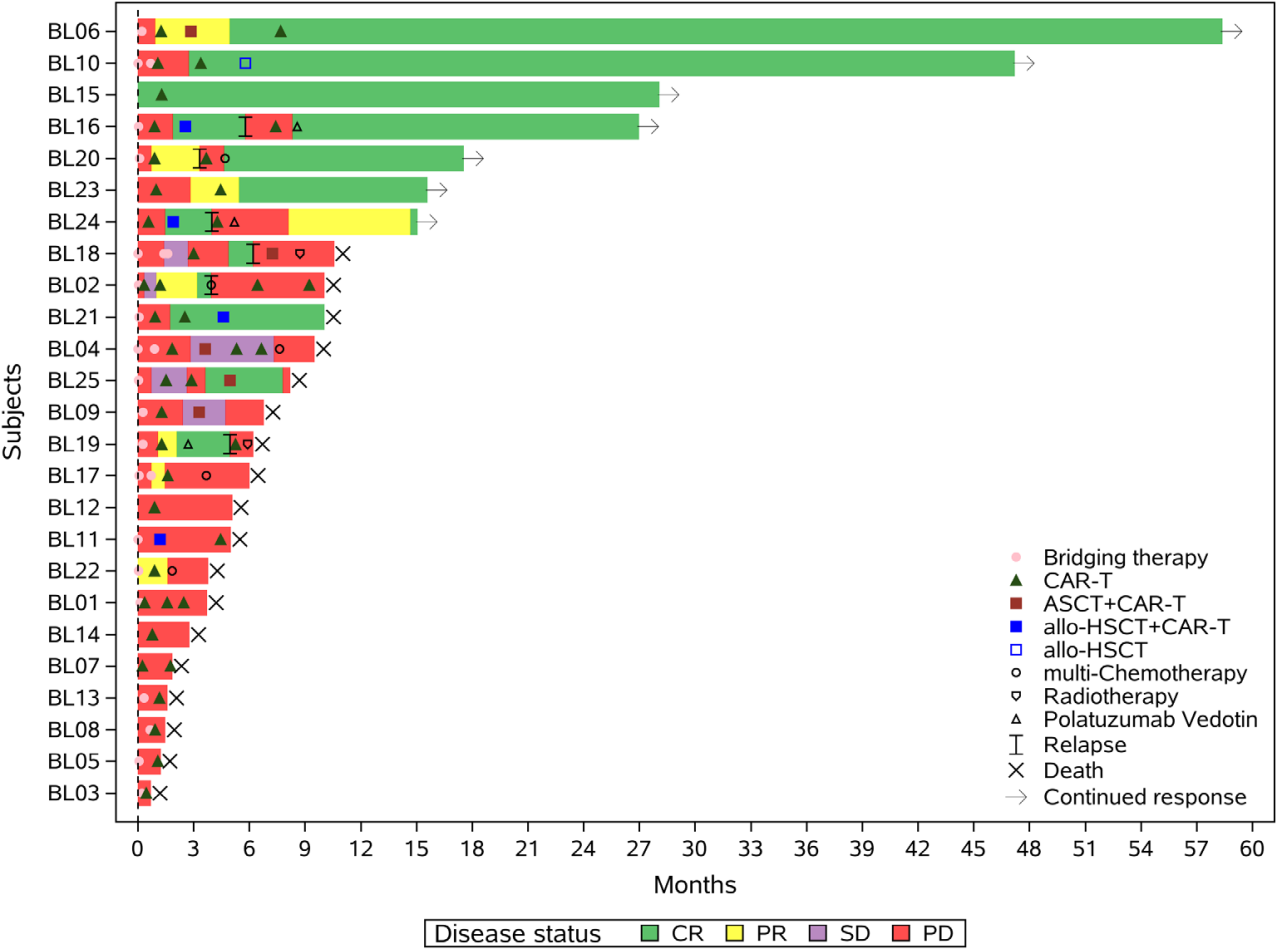
Swimmer plot illustrating the clinical response and follow-up of patients treated with the proposed regimen. Each bar represents an individual patient, with colors indicating different therapeutic interventions following CD19-directed CAR-T cell therapy, including alternative-target CAR-T products (e.g., CD20, CD22), autologous stem cell transplantation (ASCT), allogeneic hematopoietic stem cell transplantation (allo-HSCT), targeted agents, chemotherapy, and radiotherapy. The timing and sequence of interventions are shown along the timeline.allo-HSCT+CAR-T:Allogeneic Hematopoietic Stem Cell Transplantation combined with Chimeric Antigen Receptor T-Cell Therapy;ASCT+CAR-T:Autologous Stem Cell Transplantation combined with Chimeric Antigen Receptor T-Cell Therapy.

After 1 month CAR-T evaluation, 68% (17/25) received sequential consolidation therapy. The median duration of sequential treatment was 54.5 days (range: 26-127). **Figure 2** shows the swim plot of all patients, detailing the timeline and response following the first CAR-T treatment.

### AE and toxicities

All patients received CD19-targeted CAR-T, with 21 using murine-derived and 4 using human-derived constructs. The median infusion dose was 1.46 × 10^6/kg (range: 0.0126–7.22 × 10^6/kg).Among patients receiving their first CAR-T cell therapy, 80% (20/25) experienced cytokine release syndrome (CRS), and 15% (3/20) had grade ≥ 3 immune effector cell-associated neurotoxicity syndrome (ICANS), with 5/25 experiencing ICANS and 3/5 having grade ≥ 3 ICANS. The duration of CRS was 8 days (range 1-30). Pre-infusion IL-6 levels were measured in 20 patients. Although patients who developed grade ≥3 CRS (n=3) had higher IL-6 levels than those with grade ≤2 CRS (n=17)(29.52 vs. 6.61 pg/mL), the small sample size of the high-grade CRS group limits firm conclusions. Fifteen patients received corticosteroid treatment for CRS, with a median total steroid usage of 73.5 mg (range 5-243).Hematologic toxicity included 100% (25/25) incidence of grade 3–4 neutropenia, 18/25 for grade 3–4 thrombocytopenia, and 20/25 for grade 3–4 anemia. Grade ≥3 cytopenias were observed in 100% (25/25) of patients. The median time to recovery of grade ≥3 cytopenias (defined as return to grade ≤2 or baseline) was as follows:Neutropenia (NEU): median 21 days (range 7–72);Thrombocytopenia (PLT): median 18 days (range 7–62);Anemia (HGB): median 13 days (range 5–86).Granulocyte colony-stimulating factor (G-CSF) was administered in 7 patients (28%), and 17 patients (68%) received platelet or red blood cell transfusions as supportive care. These findings highlight the expected, yet significant, burden of hematologic toxicity in this population. Although supportive care was employed, the retrospective nature of the study limited the ability to fully characterize toxicity management protocols and their correlation with pre-treatment factors or clinical outcomes. While hematologic toxicity was systematically recorded, the current analysis did not assess its correlation with pre-infusion clinical predictors such as tumor burden or inflammatory cytokine levels, which warrants further investigation. Infections occurred in 36% (9/25) of patients, with 5 cases of bacterial infections, 2 fungal infections, 1 case of bacterial and COVID-19, and 1 case of bacterial and fungal infections. Among these, 4 patients experienced gastrointestinal bleeding during treatment. Four patients had seizures. There was 1 treatment-related death due to lower gastrointestinal bleeding. (**Table 2**).

**Table 2:** Adverse events after CAR-T therapy in R/R Burkitt lymphoma patients (N=25).

Adverse Event	Any	Grade 1 or 2	Grade 3	Grade 4
Hematologic event				
Neutropenia	25/25 (100%)	0	2/25 (8%)	23/25 (92%)
Thrombocytopenia	22/25 (88%)	4/25 (16%)	3/25 (12%)	15/25 (60%)
anemia	1/25 (4%)	4/25 (16%)	20/25 (80%)	
Infection				
Bacterial	5/25 (20%)			
Fungal	2/25 (8%)			
co-infection	2/25 (8%)			
gastrointestinal_bleeding	4/25 (16%)			

### Risk factors associated with the outcome

**Figure 3:**
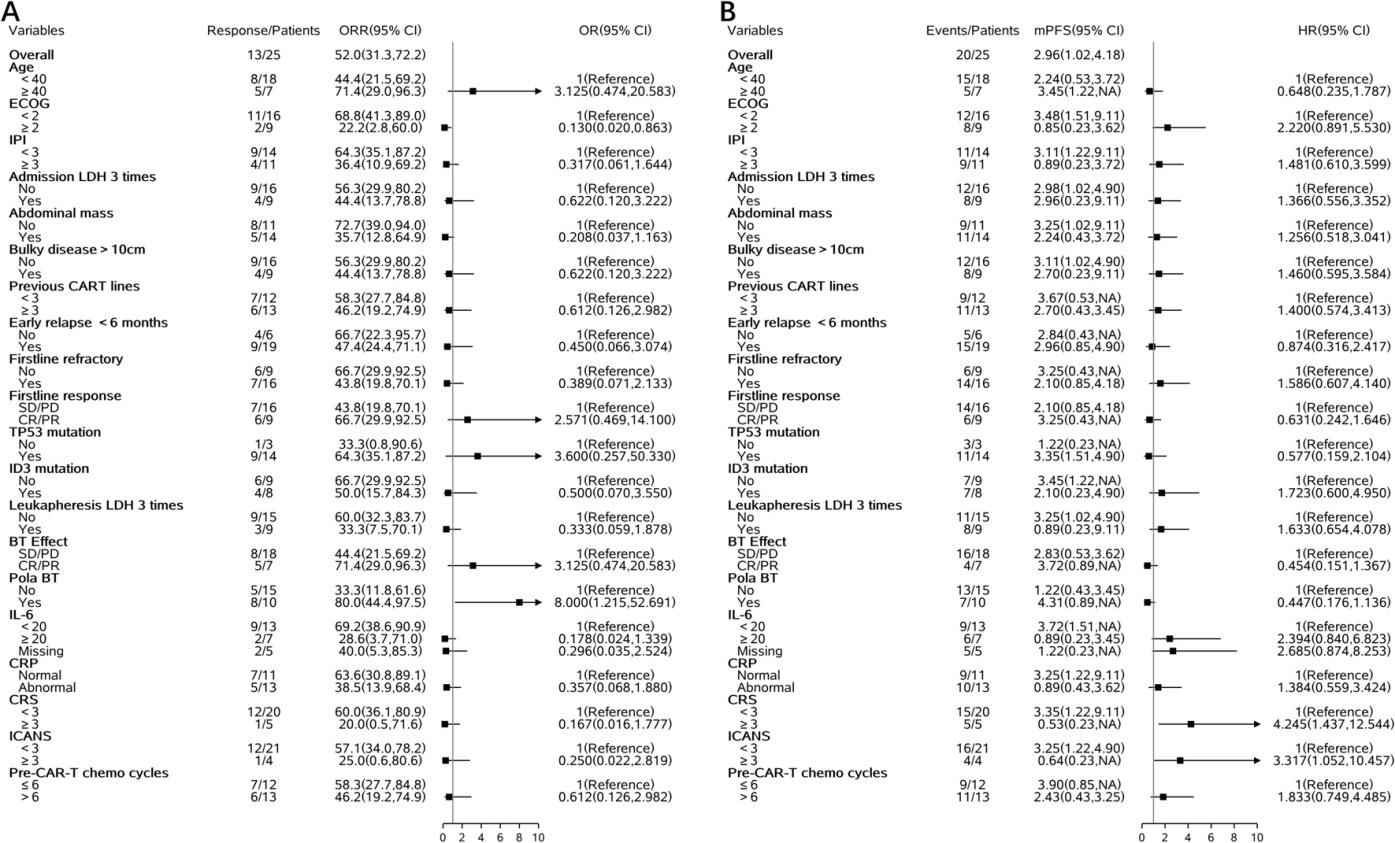
Forest plots of clinical and molecular factors associated with ORR and PFS. **(A)** ORR **(B)** PFS.

Univariate logistic regression analysis showed that several clinical factors were associated with the overall response rate (ORR) (**Figure 3**). For example, patients with ECOG score ≥2 had a significantly lower ORR compared to those with ECOG <2 (22.2% vs 68.8%), with an OR of 0.130 (95% CI: 0.020–0.863). The use of pola-based bridging therapy was associated with a higher response rate (80.0% vs 33.3%), with an OR of 8.000 (95% CI: 1.215–52.691). In addition, patients with baseline IL-6 ≥20 pg/mL had a lower ORR (28.6% vs 69.2%, OR: 0.178, 95% CI: 0.024–1.339); those with bulky disease >10 cm had a modestly lower ORR (44.4% vs 56.3%, OR: 0.622, 95% CI: 0.120–3.222); and those who had received more than three prior lines of therapy before CAR-T infusion showed a numerically reduced ORR (46.2% vs 58.3%, OR: 0.612, 95% CI: 0.126–2.982).

Univariate Cox regression analysis demonstrated that several baseline factors were also associated with progression-free survival (PFS) (**Figure 3**). Patients with ECOG score ≥2 had shorter PFS compared to those with better performance status (median PFS: 2.2 vs 3.5 months, HR: 2.220, 95% CI: 0.891–5.530). Baseline IL-6 ≥20 pg/mL was associated with inferior PFS (HR: 2.394, 95% CI: 0.840–6.823). Patients who achieved CR or PR after bridging therapy tended to have improved PFS (HR: 0.454, 95% CI: 0.151–1.367), as did those who received pola-based bridging therapy (HR: 0.447, 95% CI: 0.176–1.136). Furthermore, ID3 mutation was associated with a trend toward shorter PFS (HR: 1.723, 95% CI: 0.600–4.950).

### Biomarker analysis

**Figure 4:**
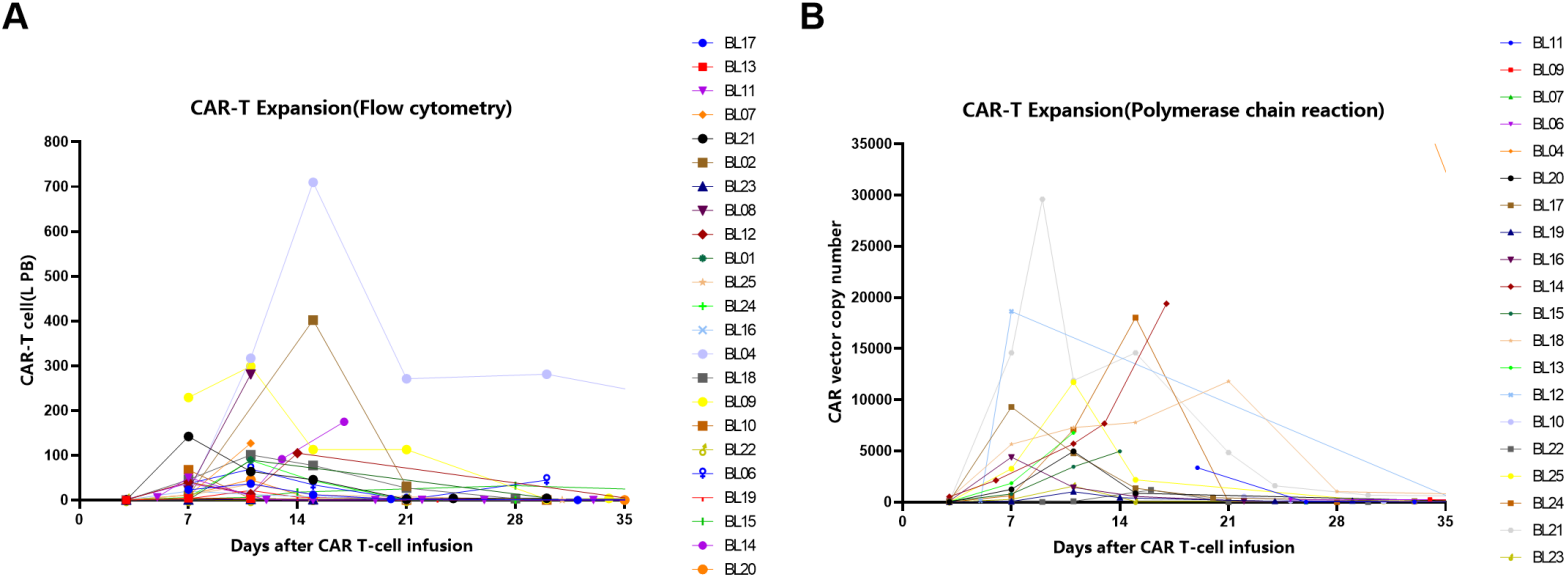
The amplification of CAR-T cells detected by Flow Cytometry method **(A)** and Polymerase chain reaction(PCR) method **(B)**.

PCR methods detected 20/25 patients, all of whom had successful amplification (20/20), with a median peak value of 4966 copies and a median time to peak of day 14.5 (range 7-34). Flow cytometry detected 23/25 patients, all of whom had successful amplification (23/23), with a median peak value of 68.1 × 10^6 and a median time to peak of day 9 (range 7-28). Additionally, 6/25 patients received pomalidomide, low-dose radiotherapy, or PD-1 stimulation for CAR-T expansion on days 7 or 14 after CAR-T infusion. Six patients received stimulatory interventions, among whom four achieved successful CAR-T cell expansion (**Supplementary Figure 1**). **Figure 4** show the amplification of CAR-T cells detected by flow cytometry method (**Figure 4A**) and PCR method (**Figure 4B**), respectively.

### Gene characteristics and survival

17 out of 25 patients underwent second-generation gene sequencing, with high-frequency mutations identified in genes including TP53, ID3, MYC, CCND3, KRAS, and FAT4. The mutation frequency, mutation types, and their association with survival were analyzed (**Tables 3**–**5**).

**Table 3:** Frequently mutated genes and their average mutation frequencies in R/R BL patients.

Gene	N	Average Frequency (%) Mean (SD)
TP53	9	71.46 (24.87)
ID3	4	64.08 (39.74)
MYC	5	80.30 (37.74)
CCND3	4	41.84 (4.24)
KRAS	2	19.79 (0.00)
FAT4	2	19.76 (0.00)

**Table 4:** Distribution of mutation types among frequently altered genes in R/R BL patients.

Gene	Mutation type							Total
	Frameshift deletion	Frameshift insertion	Nonframeshift substitution	Nonsynonymous SNV	Splicing	Stopgain	Synonymous SNV	
BCOR	1	0	0	0	0	0	0	1
DDX3X	0	0	0	1	0	0	0	1
EP300	1	0	0	0	0	0	0	1
GNA13	0	0	0	0	0	1	0	1
ID3	0	0	0	1	0	1	0	2
TET2	0	0	0	0	0	1	0	1
TPMT	0	0	0	1	0	0	0	1
ARID1A	0	0	0	0	0	6	0	6
BCOR	1	0	0	0	0	0	0	1
CCND3	0	4	0	0	0	0	0	4
CDKN2A	0	0	0	0	0	2	0	2
CREBBP	2	0	0	2	0	0	0	4
CYP2C19	0	0	0	0	0	0	2	2
DDX3X	0	0	0	1	0	0	0	1
EP300	1	0	0	0	0	0	0	1
FAT4	0	0	0	2	0	0	0	2
GNA13	0	0	0	0	2	1	0	3
ID3	2	0	1	3	0	1	0	7
JAK3	0	2	0	0	0	0	0	2
KRAS	0	0	0	2	0	0	0	2
MACF1	0	0	0	2	0	0	0	2
MAPK1	0	0	0	2	0	0	0	2
MYC	0	0	0	5	0	0	0	5
NSD1	0	1	0	0	0	0	0	1
PHF6	0	2	0	0	0	0	0	2
PRF1	0	0	0	2	0	0	0	2
SMARCA4	0	0	0	1	0	0	0	1
TCF3	0	0	0	1	0	0	0	1
TCF3x	0	0	0	1	0	0	0	1
TET2	0	0	0	0	0	1	0	1
TP53	0	0	0	7	0	2	0	9
TPMT	0	0	0	1	0	0	0	1
Total	8	9	1	35	2	16	2	73

**Table 5:** Overall Survival (OS) by Gene Status in Burkitt Lymphoma (N=17).

Gene	OS (N=17)	Yes	No	
TP53	N	14	3
	Median (95% CI)	7.22 (4.37, NA)	3.35 (0.23, NA)
ID3	N	8	9
	Median (95% CI)	5.52 (0.23, 9.70)	9.11 (3.35, NA)
MYC	N	8	9
	Median (95% CI)	8.63 (2.89, NA)	5.49 (0.23, NA)
CCND3	N	5	12
	Median (95% CI)	7.56 (2.89, NA)	6.08 (3.35, NA)
KRAS	N	2	15
	Median (95% CI)	6.23 (3.35, NA)	6.87 (3.81, NA)
FAT4	N	2	15
	Median (95% CI)	6.30 (2.89, NA)	6.87 (3.81, NA)

### Sequential consolidation therapy

**Figure 5:**
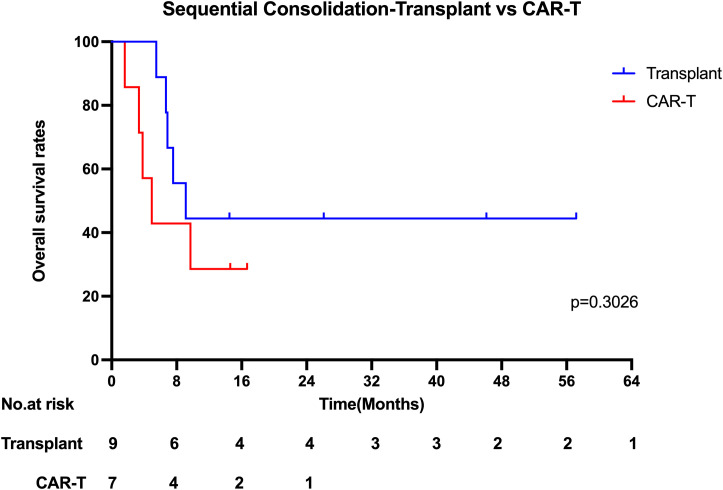
Kaplan-Meier estimates of the overall survival who underwent sequential Consolidation therapy.

Among the 25 patients, 16 received sequential consolidation therapy, while 9 did not. The median overall survival (OS) was significantly longer in the sequential consolidation group compared to the non-consolidation group (8.34 vs. 2.01 months; p = 0.0033). Similarly, the median progression-free survival (PFS) was 3.54 months in the consolidation group and 0.53 months in the non-consolidation group (p = 0.0378).With a median follow-up of 26.10 months (range, 14.50–57.17 months), 6 out of 16 patients who received sequential consolidation therapy remained alive, compared to only 1 out of 9 in the non-consolidation group. Among patients receiving sequential consolidation, 9 underwent hematopoietic stem cell transplantation (HSCT), and 7 received additional CAR-T cell therapy. In the HSCT group, 4 of 9 patients were alive at the last follow-up. Specifically, 5 patients underwent autologous HSCT (1/5 survived), and 4 received allogeneic HSCT (3/4 survived). In the CAR-T consolidation group, 2 of 7 patients remained alive. The median OS for CAR-T consolidation and transplantation consolidation were 4.93 months and 9.11 months (**Figure 5**), respectively (p = 0.3026). Despite this, a higher number of patients who underwent bone marrow transplantation consolidation achieved long-term survival, suggesting that it may contribute to improved long-term outcomes.

### Individualized sequential consolidation and long-term survivors

Of the 9 without sequential therapy, only one (BL15) achieved CR during bridging and remained disease-free for 2+ years post-CD19-CAR-T without consolidation. The other 8 patients experienced PD, including 6 who were unable to receive further treatment and 2 who underwent 2 cycles of POLA-based chemotherapy but showed continued progression. The median survival for these 8 patients was 1.27 months (range 0.23–4.37). **Figure 2** shows individualized therapies for patients with sequential treatments, including alternative-target CAR-T, ASCT, allo-HSCT, targeted agents, multi-agent chemotherapy, and radiotherapy. Seven patients remain alive:BL06 achieved PR post-CD19-CAR-T, followed by ASCT and CD22-CAR-T, and later received CD20-CAR-T at 6 months. The patient has been in remission for 5 years.BL10 achieved CR post-CD19-CAR-T, followed by CD22-CAR-T and allo-HSCT at 5 months, and remains in remission.BL15 achieved CR post-CD19-CAR-T without further treatment and has been disease-free for 26 months.BL16 achieved CR post-CD19-CAR-T and underwent allo-HSCT with CD19&CD22-CAR-T. Despite initial remission at 1–2 months, relapse occurred at 3 months. The patient received CD20-CAR-T followed by 11 cycles of POLA maintenance therapy and has been disease-free for 22 months.BL20 achieved PR post-CD19-CAR-T, relapsed, and achieved CR post-CD20-CAR-T. The patient then received 2 cycles of R-ICE and has been disease-free for 16 months.BL23 achieved PR post-CD19-CAR-T, followed by CD20-CAR-T, and has been disease-free for 14 months.BL24 achieved remission post-CD19-CAR-T, underwent allo-HSCT with CD20-CAR-T, but progressed at 104 days. The patient subsequently received CD19&CD22-CAR-T and achieved remission for 14 months.

## Discussion

Refractory relapsed Burkitt lymphoma (R/R BL) remains a clinical challenge,with limited efficacy from conventional chemotherapy and targeted agents. In our cohort of 25 patients, bridging chemotherapy showed poor effectiveness, with only 21% achieving a partial response. This highlights the chemo-resistance of this population. After the first CAR-T infusion, the overall response rate (ORR) was 52%, and the complete response (CR) rate was 28%. Although CAR-T therapy provided a certain degree of tumor control, durable remission was limited.

Our findings align with recent data from Samples et al. (2025), who reported on adult patients with R/R BL treated with CAR-T therapy. Their study demonstrated an ORR of 58% and a CR rate of 41.9%, with a median progression-free survival (PFS) of 2.3 months and a median overall survival (OS) of 6.0 months. These results, comparable to ours, underscore the limited durability of response and high rate of early relapse following CAR-T therapy in this setting (25). Additionally, case reports and small cohorts have shown that early responses to CAR-T in both adult and pediatric R/R BL can be encouraging, but long-term remission is rare without consolidation (18–20, 26, 27).

Consistent with this, Liu Y, et al. analyzed outcomes of 21 adult R/R BL patients receiving CAR-T therapy with or without autologous hematopoietic stem cell transplantation (HCT). The group receiving CAR-T therapy combined with autologous HCT (n=8) had an ORR of 87.5% and significantly improved 1-year OS and PFS compared to the CAR-T alone group (n=13) (p = 0.014 and p = 0.045, respectively), suggesting that combination strategies may enhance long-term disease control (34).Specific follow-up treatment strategies should be tailored according to changes in the patient’s condition. These strategies may include allogeneic hematopoietic stem cell transplantation, alternative targeted CAR-T therapies, or autologous hematopoietic stem cell transplantation, aiming to reduce the risk of relapse. Early initiation of consolidation therapy could play a crucial role in maintaining long-term remission (33). However, our statistical analysis did not show a significant difference between CAR-T consolidation and transplantation consolidation. The median OS for CAR-T consolidation and transplantation consolidation were 4.93 months and 9.11 months, respectively (p = 0.3026). Despite this, a higher number of patients who underwent bone marrow transplantation consolidation achieved long-term survival, suggesting that it may contribute to improved long-term outcomes. Given the limited sample size, further studies with larger cohorts are required to confirm the potential benefits of transplantation.

We conducted genetic mutation analysis on 17 patients who underwent next-generation sequencing and found common mutations, including TP53, ID3, MYC, and CCND3. These mutations may have some impacts on treatment response and prognosis. In this cohort of relapsed/refractory Burkitt lymphoma patients, the prognostic impact of mutations in TP53, MYC, ID3, CCND3, KRAS, and FAT4 remains unclear. Due to the small sample size in this study, further prospective research is needed to validate the relationship between these genetic mutations and prognosis (28, 29). The safety of CAR-T cell therapy is a key consideration in clinical application. In our study, 84% of patients experienced cytokine release syndrome (CRS), with 20% having grade ≥ 3. Immune effector cell-associated neurotoxicity syndrome (ICANS) occurred in 24% of patients, with 16% being grade ≥ 3. Regarding hematologic toxicity, 100% of patients experienced neutropenia, 18 patients had grade 3–4 thrombocytopenia, and 20 patients had grade 3–4 anemia. Severe CRS and ICANS appeared to be negatively associated with survival. Grade ≥3 CRS was associated with shorter PFS (HR: 4.245; 95% CI: 1.437–12.544) and a trend toward lower ORR (OR: 0.167; 95% CI: 0.016–1.777). Similarly, grade ≥3 ICANS was associated with inferior PFS (HR: 3.317; 95% CI: 1.052–10.457) and a numerically lower ORR (OR: 0.250; 95%).CI: 0.022–2.819 ≤ These toxic reactions emphasize the need for strict monitoring and timely intervention for patients. We recommend a multidisciplinary team approach to manage CAR-T-treated patients to address potential severe adverse reactions (30).In addition to the common side effects mentioned, the risk of infection following CAR-T treatment is also a major safety concern. Because CAR-T cell therapy significantly suppresses the immune system, patients may face an increased risk of bacterial, fungal, and viral infections. Therefore, prophylactic anti-infective treatment and close infection monitoring are essential (31, 32). Several baseline factors—including ECOG performance status ≥2, elevated baseline IL-6 levels, and failure to achieve remission following bridging therapy—were associated with inferior outcomes after CAR-T cell treatment. In contrast, bridging regimens incorporating polatuzumab vedotin (POLA) demonstrated a trend toward improved response rates and progression-free survival (PFS). Incorporating POLA into bridging strategies may enhance pre-CAR-T disease control, thereby increasing the likelihood of achieving remission following CAR-T infusion.

In addition to these baseline indicators, prior treatment intensity may also impact CAR-T efficacy. The median number of prior chemotherapy cycles was 7 (range, 3–16). We hypothesized that intensive early-line treatment might compromise subsequent CAR-T efficacy. Patients were stratified by whether they had received >6 cycles of chemotherapy before CAR-T infusion. Among them, 13 of 25 (52%) received >6 cycles. This group showed a trend toward shorter progression-free survival (HR 1.833, 95% CI: 0.749–10.457) and a lower overall response rate (OR 0.612, 95% CI: 0.126–2.982).

To further improve outcomes, future strategies may involve the use of multi-targeted CAR-T products (e.g., CD19/CD22/CD20), innovative CAR designs, expedited manufacturing platforms, and integration with consolidative or maintenance therapies. The identification of predictive biomarkers—such as baseline IL-6, ECOG status, and molecular mutations—may facilitate more accurate risk stratification and individualized treatment planning. In addition, optimizing pre-CAR-T treatment regimens—such as minimizing overly intensive frontline chemotherapy—may help preserve T-cell fitness and enhance CAR-T efficacy. Given the aggressive biology and propensity for early relapse in BL, intensified post-CAR-T management should be a focus of future clinical investigations.

## Conclusion

CAR-T therapy shows promising activity in relapsed/refractory Burkitt lymphoma, but its effectiveness is limited by short response duration. High-risk features may predict poor outcomes, and a higher number of long-term survivors were observed in patients who received transplant sequential consolidation. However, due to the small sample size, larger studies are needed to validate these findings.

## Data Availability

The original contributions presented in the study are included in the article/**Supplementary Material**. Further inquiries can be directed to the corresponding authors.
