# Supplementary figures and images for: CAR T- cell therapy provides an opportunity for further consolidation treatment for relapsed or refractory adult Burkitt lymphoma patients
Source: Front Oncol. 2025 May 23;15:1566938. doi: 10.3389/fonc.2025.1566938 (PMC12141207; doi:10.3389/fonc.2025.1566938)

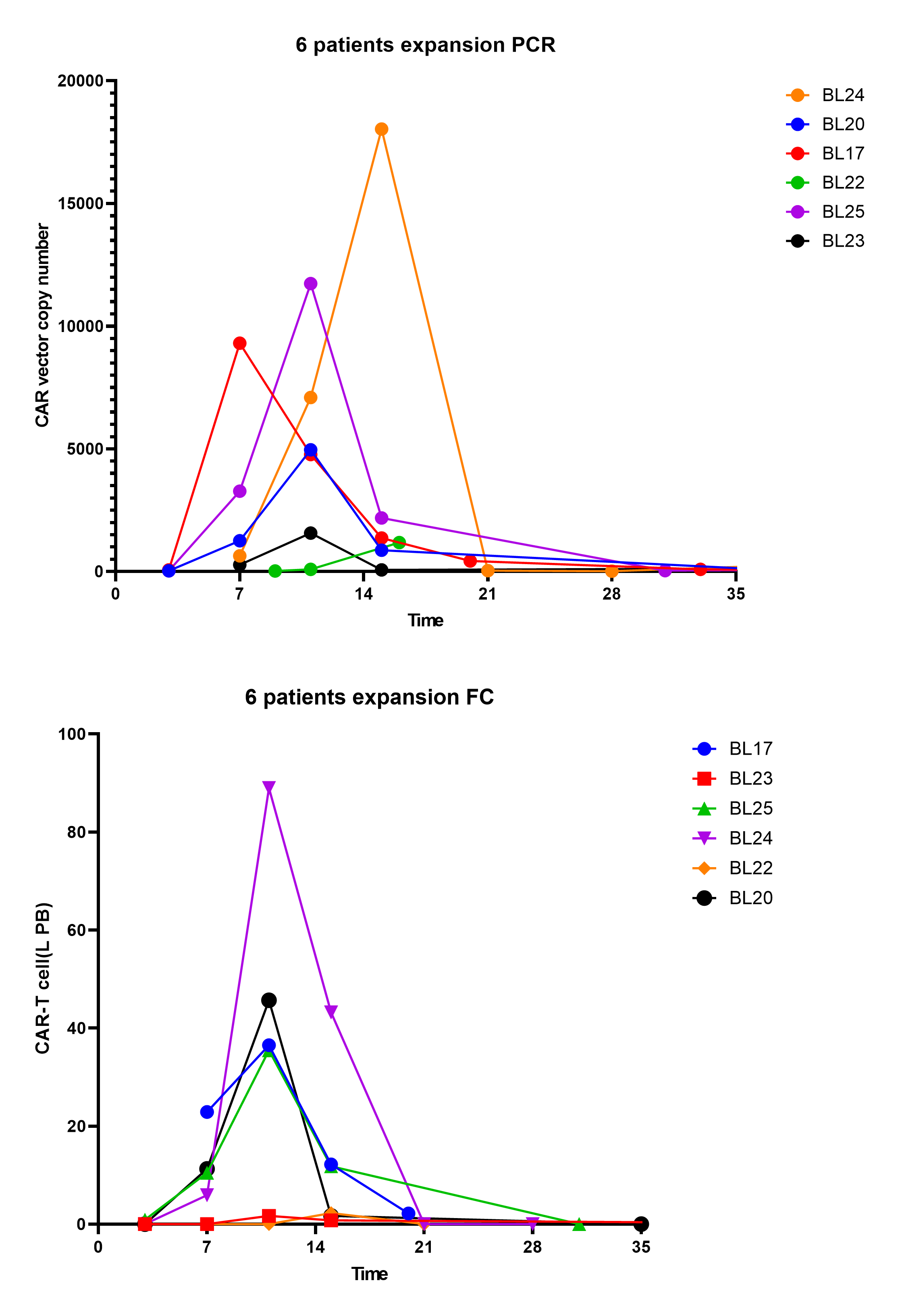

Supplement: Supplementary file 1 [file Image1.tif]
